# Tracheoesophageal Fistula due to a Damaged Tracheal Stent

**DOI:** 10.1155/2014/926387

**Published:** 2014-05-05

**Authors:** Masahiro Kimura, Yoshiyuki Kuwabara, Hideyuki Ishiguro, Tatsuya Tanaka, Hiromitsu Takeyama

**Affiliations:** Department of Gastroenterological Surgery, Nagoya City University Graduate School of Medical Sciences, 1 Kawasumi, Mizuho-cho, Mizuho-ku, Nagoya 467-8601, Japan

## Abstract

We describe the management of a tracheoesophageal fistula due to a damaged tracheal stent, which was first inserted to treat tracheal stenosis. A 29-year-old woman with a history of treated epilepsy had a seizure and suffered from smoke inhalation during a fire. Breathing difficulties appeared and gradually worsened; consultation was obtained two years afterward. After undergoing a thorough examination, the patient was diagnosed with tracheal strangulation. A noncovered, metallic stent was inserted. When the patient was 37 years old, she was admitted to our hospital for the treatment of a tracheoesophageal fistula. We diagnosed it as a tracheoesophageal fistula due to the collapse of the damaged tracheal stent toward the esophageal side, and we decided to perform a mediastinal tracheostomy. Granulation may be formed in the circumference of a stent that has been present for a prolonged period, and removal of the stent may become difficult. This case suggests that insertion of a noncovered, metallic stent is contraindicated for a benign disease.

## 1. Case Report

A 37-year-old woman was admitted to our hospital for the treatment of a tracheoesophageal fistula. Medical history revealed that she had epilepsy and took anticonvulsant medication continuously from the age of 10 years. When the patient was 29 years old, she had an epileptic seizure when a fire occurred, and she suffered from smoke inhalation. Chronic coughing and breathing difficulties appeared a few months after the fire. The symptoms gradually worsened, and hospital consultation was obtained two years afterward. As a result of a thorough examination, she was diagnosed as having breathing difficulty due to tracheal strangulation. A noncovered, metallic Ultraflex Tracheobronchial Stent (Boston Scientific Corporation, Natick, MA) was inserted approximately 2 years after the fire. Since there was no evidence of congenital disease, the cause of tracheal stenosis was attributed to smoke inhalation based on her history. Although her condition improved after stent insertion, repeated bronchoscopic medical treatment was carried out for modification of the stent and granulation formation.

One year after stent insertion, the patient experienced postprandial obstruction. Since the airway narrowing had worsened, she returned to the hospital. An attempt was made to remove the stent completely. However, it was possible to extract only part of the stent due to adhesion of the stent and the trachea. The stent had been inserted in the trachea on the proximal side while the tracheotomy was performed. Subsequently, food was discharged from the tracheotomy, and a tracheoesophageal fistula was diagnosed. For this reason, nutrition was provided primarily via the gastrostoma.

**Figure 1:**
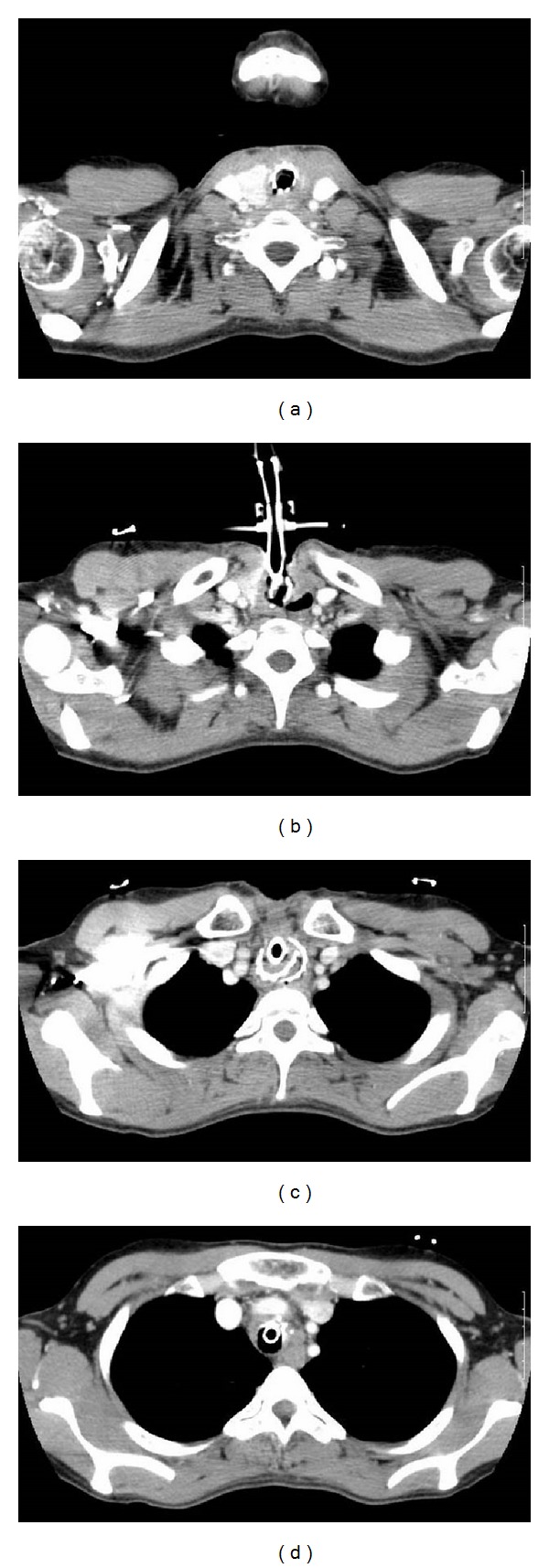
Computed tomography scans. (a) The stent was inserted proximally to the tracheotomy. (b) The tracheotomy was shown on CT. (c) The part of the destroyed stent that was distal from the tracheotomy was not circular. (d) The lower end of the stent was shown on the CT scan.

**Figure 2:**
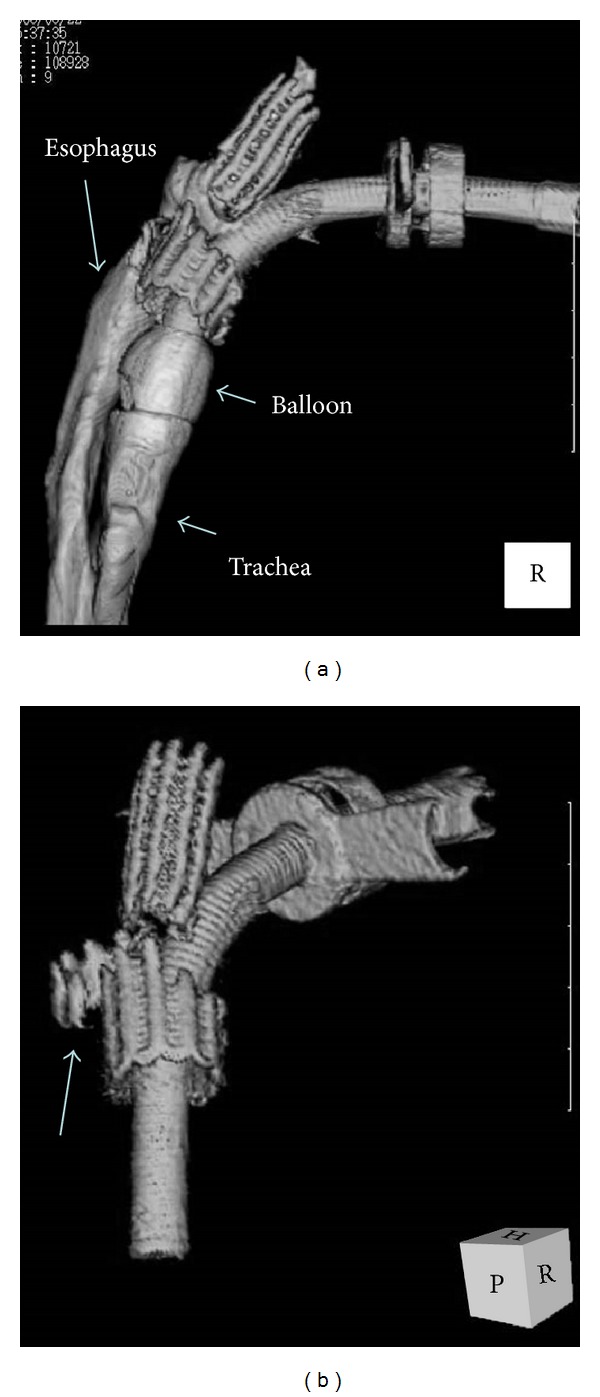
Reconstruction pictures on computed tomography. (a) The spatial relationship of the trachea, esophagus, stent, and tracheal tube was shown on CT. (b) The destroyed stent with a section existing outside of the trachea was also shown on CT (arrow).

When the patient was 37 years old, she was admitted to our hospital for a procedure to remove the stent and close the tracheoesophageal fistula. Computed tomographic (CT) scans showed strangulation of the stent on the proximal side from the tracheotomy. The part of the stent distal from the tracheotomy was not a circular form. The deepest part of the stent was inferior to the left innominate vein (Figure 1). Based on the reconstruction picture of the CT, the stent was fractured, and one section was positioned posteriorly and to the left of the trachea (Figure 2).

**Figure 3:**
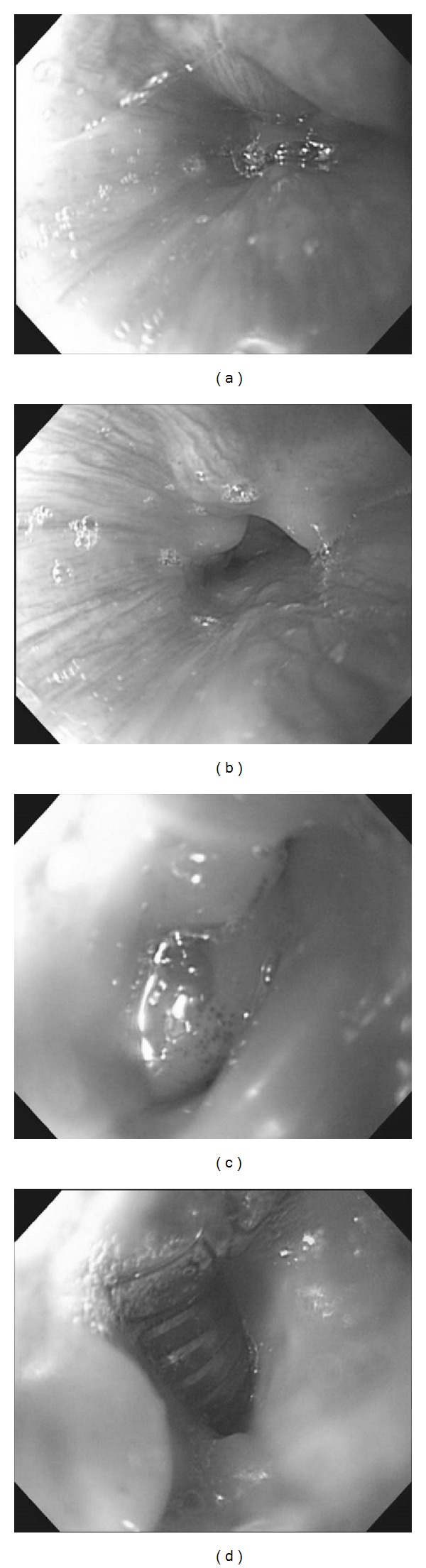
Esophagoscopy. (a) The esophageal orifice was visible on esophagoscopy. (b) Strangulation in the cervical esophagus was present. (c) Granulation tissue had developed. (d) A defect in the esophageal wall was present, and the tracheal tube was visible.

**Figure 4:**
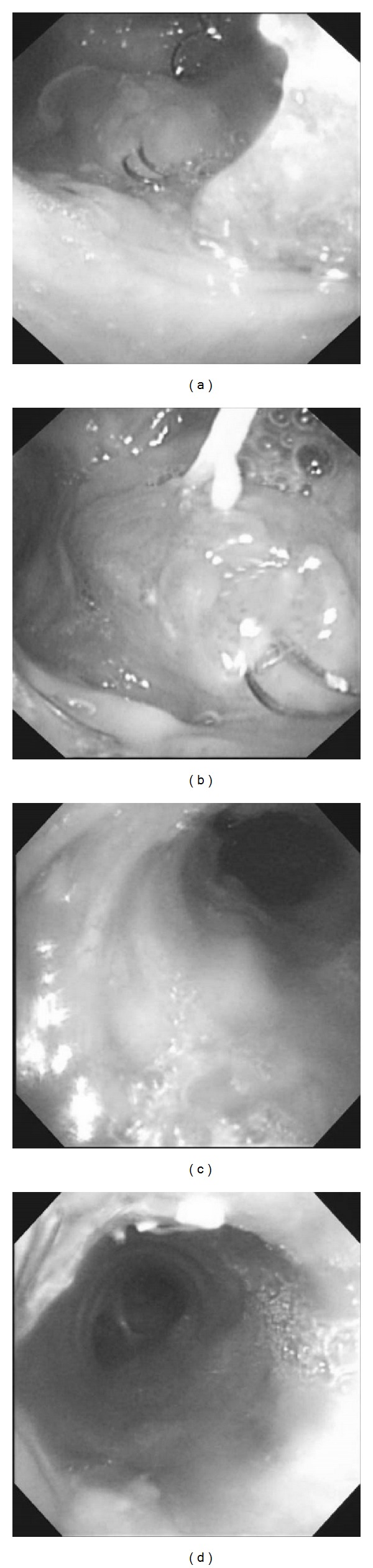
Bronchoscopy. (a) The top of the stent and the esophageal orifice were visible on bronchoscopy. (b) Food was emerging from the side of the granulation. (c) The area inside the stent was shown. (d) The trachea peripheral to the stent was also visible on bronchoscopy.

Esophagoscopy revealed the following: stenosis in the cervical esophagus, granulation in the distal side, and the tracheal tube in the distal side (Figure 3). When the tracheal tube was removed, bronchoscopy revealed the covered stent. Granulation was present directly under the tracheal hole, and food emerged from the side of the granulation. Abnormalities did not extend to the peripheral trachea from the stent (Figure 4). When esophagography was performed, leakage into the trachea was apparent.

We diagnosed it as a tracheoesophageal fistula due to the fact that the damaged tracheal stent collapsed to the esophageal side, and we decided to operate. The patient was intubated from the tracheotomy and underwent right thoracotomy at the fifth intercostal space. We planned to observe the length of the damaged trachea through the intercostal thoracotomy incision. The operation was due to be stopped when the remaining trachea was shortened. The thickening of pleural membranes and adhesions were observed in the upper mediastinum. First, the upper esophagus was peeled away from the trachea. Adhesions were intense, so that they went to the proximal side. We could peel the esophagus only to the lower end of the stent but no further. At this point, we judged that the respiratory tract reconstruction would require an anterior mediastinal tracheostomy [1].

**Figure 5:**
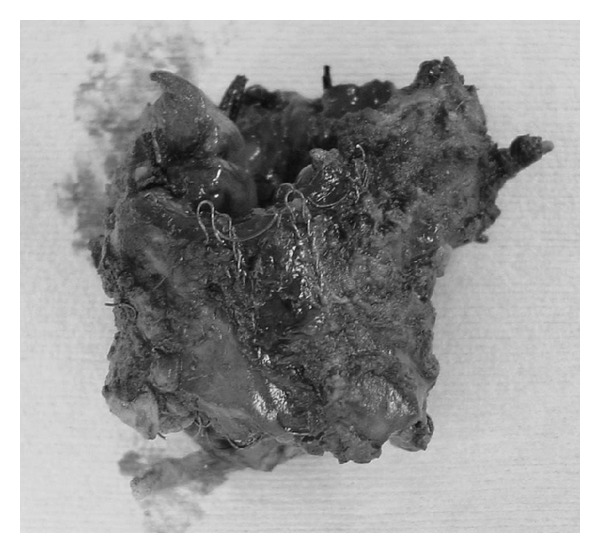
The stent was coiled around the extracted trachea.

**Figure 6:**
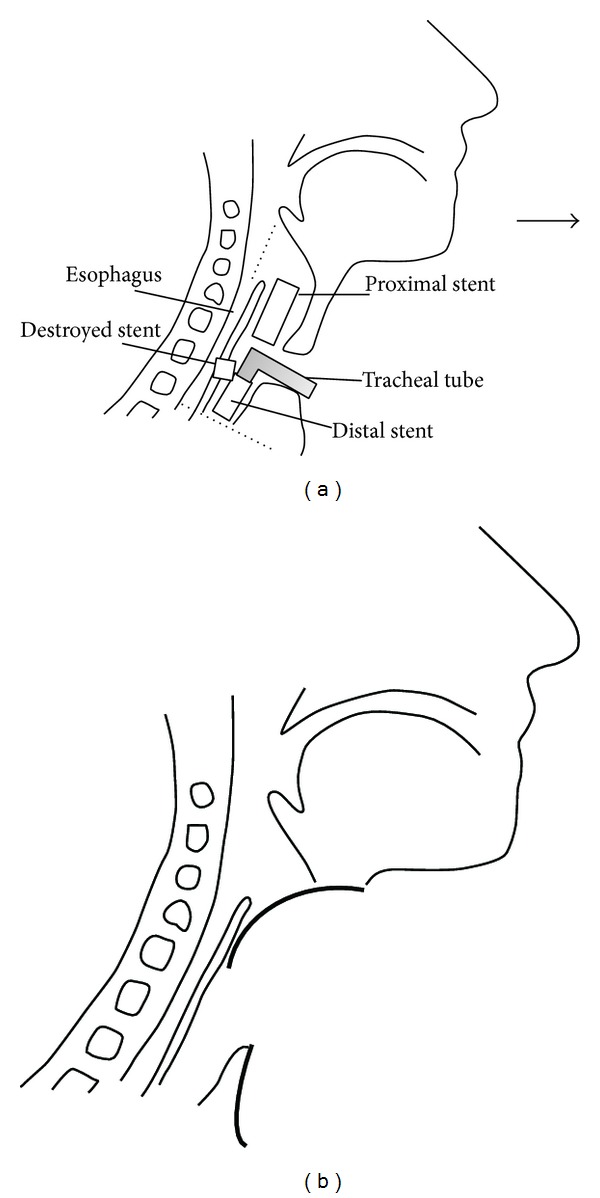
The schema of the tracheal excision range is represented by the dotted line on the left diagram. The postoperative anatomy, after excision of the trachea and removal of the destroyed stent, is shown in the right diagram.

A U-shaped incision was made just above the isthmus of the thyroid, and a vertical midline incision was made from the bottom of the U incision to the level of the third rib. The sternocleidomastoid muscles were divided at their origins. The pectoralis muscles were reflected off the chest wall bilaterally to expose the ribs and their costal cartilage. With a sternal saw, the anterior thoracic breastplate was resected, including the medial third of the clavicles, the medial segments of the first and second costal cartilages, and the upper third of the sternum to the upper edge of the third rib. The ample removal of the anterior thoracic wall allowed excellent exposure of the thoracic inlet and access to the cervicothoracic esophagus. The trachea was divided gently from the esophagus, and the trachea and the damaged stent were extracted (Figure 5). The length of the distal trachea was 5 cm. The damaged esophageal wall was sutured by about 1/2 rounds layer to layer. The trachea was transposed inferiorly and between the superior vena cava and aortic arch. The end of the trachea was sutured to the skin overlying the resected sternum (Figure 6). The patient's postoperative progress has been good, and her hospital stay lasted for 45 days after the operation. After leaving the hospital, the patient was seen in follow-up at 6 months after discharge, and she has been able to take meals sufficiently.

## 2. Discussion

The medical treatment for airway narrowing includes laser surgery, balloon extension, a stent, a tracheotomy, and tracheal plastic surgery. The treatment is chosen according to the cause and the condition of the stenosis. Stents, either silicon or metal, are placed so that the trachea and bronchus may remain expanded. Silicon stents, such as the Dumon Stent (Boston Medical Products, Westborough, MA), have projections for the prevention of migration.

The cases for which stent insertion is needed include those with

- pressure on the respiratory tract caused by malignant diseases, such as lung cancer, esophageal cancer, or thyroid cancer [2, 3];
- strangulation due to a benign disease as in this example, including smoke inhalation and bronchomalacia [4, 5];
- bronchoesophageal fistula.

However, the following complications may occur after placement of an airway stent for benign disease: stent migration, sputum retention, and granulation tissue at the ends of the stent. In this case, the noncovered Ultraflex Tracheobronchial Stent was inserted in the tracheal strangulation. A metal stent is made so that insertion can be relatively easy and so there is relatively little sense of incongruity after insertion compared with a stent made from silicon. However, granulation is formed in the circumference of a stent that remains in place for a prolonged period, and removal of the stent may become difficult. This is one of the precautions that should be considered prior to stent insertion for a benign disease. Since there is no cover, it may develop erratic adhesions to the trachea with time. If a stent becomes damaged and deformed, it may become difficult to remove and may cause damage to an adjacent tissue. In this case, the tracheal epithelium had stretched to the inner aspect of the stent. Moreover, the area of the stent that was damaged by the removal operation underwent retroflexion to the esophageal side, causing esophageal injury and a tracheoesophageal fistula. Although the damaged esophagus was restored with sutures without excising the trachea, the stent could not be removed; therefore, the mediastinal tracheostomy was extended.

The findings in this case should alert surgeons to the fact that benign strangulation is a contraindication, not an indication, for therapy with a noncovered, metallic stent. Thus, the remaining options for medical treatment for strangulation due to benign diseases include laser ablation, surgical resection, balloon extension, a tracheotomy, tracheal plastic surgery, or use of a covered metallic or silicone stent.

## 3. Conclusion

This case suggests that insertion of a noncovered, metallic stent is contraindicated for benign disease. A covered or silicone stent must be selected for benign cases.
